# Testing the Weiss-Harter-Model in a prospective study design: the importance of perceived social support for youth physical activity

**DOI:** 10.1007/s12662-023-00883-w

**Published:** 2023-04-29

**Authors:** Julian Fritsch, Carina Nigg, Claudia Niessner, Steffen Schmidt, Alexander Woll, Darko Jekauc

**Affiliations:** 1 Institute of Sports and Sports Science, Karlsruhe Institute of Technology, 76131 Karlsruhe, Germany; 2 University of Bern, Bern, Switzerland

**Keywords:** Enjoyment, Moderate-vigorous physical activity, Self-esteem, Perceived competence, Adolescents

## Abstract

To counteract low physical activity levels in children and adolescents, it is crucial to understand the relevant psychological processes that can promote physical activity in this age group. The Weiss-Harter model focuses on self-esteem as a central construct for physical activity promotion in youth, which mediates the effects of perceived competence and perceived social support on enjoyment and physical activity. However, in two cross-sectional studies, an adapted model was found to have a better model fit in which perceived social support has additional direct effects on physical activity and enjoyment. The purpose of the present study was to compare the original Weiss-Harter model and the adapted model in a prospective study design. Data were based on two assessment waves of the German Motorik-Modul-Study involving 1107 participants (603 female) with a mean age of 13.98 years (*SD* = 2.03). Participants filled out questionnaires on perceived competence, perceived social support, self-esteem, enjoyment, and moderate-vigorous physical activity (MVPA) during the first assessment. MVPA was again assessed about five years later allowing to test whether the models could predict (1) future MVPA and (2) the difference of MVPA from the first to the second assessment. For both research questions, the original Weiss-Harter model (Model 1a: *χ*^2^ = 812.44; *df* = 95; *p* < 0.01; CFI = 0.905; RMSEA = 0.083; Model 2a: χ2 = 755.29; *df* = 95; *p* < 0.01; CFI = 0.910; RMSEA = 0.079) had a worse fit than the adapted model (Model 1b: *χ*^2^ = 512.19; *df* = 93; *p* < 0.01; CFI = 0.943; RMSEA = 0.065; Model 2b: *χ*^2^ = 513.25; *df* = 93; *p* < 0.01; CFI = 0.943; RMSEA = 0.064). The results of this study highlight the role of perceived social support for youth MVPA.

Physical activity in children and adolescents is associated with a variety of physical and mental health outcomes. For example, higher levels of physical activity have been associated with a lower risk of developing depressive symptoms (Kandola, Lewis, Osborn, Stubbs, & Hayes, 2020), a lower risk of becoming obese (Ekblom-Bak, Ekblom, Andersson, Wallin, & Ekblom, 2018), and enhanced bone mass (Mantovani et al., 2018). Although there is clear evidence for the benefits of regular physical activity, research shows that physical activity often declines during adolescence (Finne, Bucksch, Lampert, & Kolip, 2011; Kandola et al., 2020). Together with the finding that the level of physical activity in youth predicts the level of physical activity later in life (Telama et al., 2014), the decrease in physical activity points to the importance of understanding the factors that contribute to the maintenance of physical activity at a young age.

In exercise psychology, physical activity has been studied from different perspectives. Traditionally, based on social cognitive approaches (e.g., Ajzen, 1991), much research used social-cognitive constructs such as intention or self-efficacy to explain differences in physical activity (e.g., Hagger, Chatzisarantis, & Biddle, 2002). Consistent with dual-process approaches (e.g., Brand & Ekkekakis, 2018; Strobach, Englert, Jekauc, & Pfeffer, 2020) another stream of research focused on the role of affective processes related to physical activity (e.g., Rhodes & Kates, 2015). The focus on affective processes is based on the idea that individuals are more likely to engage in behaviour associated with positive affective responses and avoid behaviours associated with negative affective responses (Brand & Ekkekakis, 2018). Because these theoretical approaches have primarily focused on the physical activity of adults, the question arises to what extent they can be transferred to children and adolescents. The brain development of youths implies that they differ substantially from adults in their cognitive processes such as working memory and response inhibition (Luna, Padmanabhan, & O’Hearn, 2010). Moreover, children and adolescents usually have less autonomy over their behaviour and rely more on the support of caregivers (Rosenkranz, Ridley, Guagliano, & Rosenkranz, 2021). It is also argued that attempts to increase physical activity among children and adolescents should focus less on the consequences of physical activity (e.g., increasing the awareness of health-related outcomes), but rather on the psychological processes that drive them to be physically active (Weiss, 2000). These aspects are taken into account by the Weiss-Harter model (Weiss, 2000), which focuses explicitly on youth physical activity and provides the theoretical framework of the present study.

By adapting Harter’s (1987) model of self-esteem to the domain of physical activity, the core assumption of the Weiss-Harter model is that self-esteem mediates the effects of perceived competence and perceived social support on enjoyment and physical activity (see Fig. 1 in the result section as a graphic representation). In this sense, the Weiss-Harter model encompasses constructs such as enjoyment, perceived social support, or perceived competence, which are also relevant in dual-process approaches and social-cognitive approaches, while additionally emphasising the role of the self.

Contemporary theoretical approaches on the self are often based on the model of Shavelson, Hubner, and Stanton (1976), in which the self is conceptualized in a hierarchical and multidimensional structure. Considering the general self-concept at the apex of this structure, the physical self-concept is one of its main domains, which in turn is subdivided into more specific domains, such as the perceptions of one’s physical ability or physical appearance. While the general self-concept and its domains usually include descriptions of oneself, self-esteem carries an evaluative component involving an assessment of one’s worth (Harter, 1987). The extent to which individual domains of the self-concept influence one’s self-esteem depends on the importance the individual attaches to the specific domain (Fox & Wilson, 2008). The psychological construct of self-esteem has been addressed by various theoretical approaches related to physical activity, such as the exercise and self-esteem model (Sonstroem & Morgan, 1989), the skill development model, the self-enhancement model, or the reciprocal effects model (for a comparison of the last three see Marsh, Papaioannou, & Theodorakis, 2006), and is also the central psychological construct in the Weiss-Harter model (Weiss, 2000).

According to the Weiss-Harter model, self-esteem mediates the effects of perceived competence and perceived social support on physical activity and enjoyment (Weiss, 2000). The positive direct effect of self-esteem on physical activity is based on the idea that individuals are motivated to pursue situations in which they can effectively demonstrate their abilities and is in line with the aforementioned self-enhancement model (e.g., Fox & Wilson, 2008). This assumption is generally supported in the literature (e.g., Adachi & Willoughby, 2014; deJonge, Mackowiak, Pila, Crocker, & Sabiston, 2019; for a narrative review see Fox & Wilson, 2008). At the same time, consistent with the reciprocal effects model (Marsh et al., 2006), it is important to emphasize that studies suggest that the association between self-esteem and physical activity is bi-directional (e.g., Garn et al., 2019; Trautwein, Gerlach, & Ludtke, 2008; Wagnsson, Lindwall, & Gustafsson, 2014). These findings indicate that higher self-esteem leads to more physical activity, while at the same time more physical activity leads to higher self-esteem.

Perceived competence is another psychological construct in the Weiss-Harter model (Weiss, 2000). Considering the hierarchical model of Shavelson et al. (1976), the physical self-concept as a subdomain of the general self-concept often includes perceptions of competence (Fox & Wilson, 2008). In the context of physical activity, these perceptions comprise different aspects such as strength, endurance, or coordination (Marsh & Redmayne, 1994). In a meta-analysis that focused on the association of various forms of physical self-concept and physical activity, perceived competence had the strongest association with physical activity (Babic et al., 2014). The association between perceived competence and physical activity is consistent with the assumption that high perceived competence is associated with a higher expectation of success, which makes it more likely that a behaviour will be maintained persistently (Harter, 1978). Moreover, in line with the Weiss-Harter model, perceived competence has been shown as a source of higher global self-esteem in youth (Ebbeck & Weiss, 1998).

Perceived social support refers to social sources that may support physical activity (Weiss, 2000). For children and adolescents, parents constitute an important social element in their socialization. Pointing to the importance of role modelling, parents’ physical activity level has been shown to be positively associated with the level of their children (Garriguet, Colley, & Bushnik, 2017). Parents may also indirectly influence their children by providing opportunities and encouragement for physical activity (Tate et al., 2015). Besides parents, peers also have been shown to have an impact on adolescents’ physical activity (Fitzgerald, Fitzgerald, & Aherne, 2012). In particular, perceived peers’ social support has been shown to influence physical activity as well as their enjoyment and self-efficacy (Silva, Lott, Mota, & Welk, 2014).

The final construct in the Weiss-Harter model (2000) is enjoyment. Enjoyment as a positive emotion linked with experiences of pleasure or fun has been consistently associated with physical activity in youth (for a meta-analysis see Nasuti & Rhodes, 2013). Because enjoyment of physical activity may be an important buffer against the declines in physical activity during adolescence (Haas, Yang, & Dunton, 2021), understanding the factors associated with enjoyment may be helpful both from theoretical as well as applied perspectives. According to the Weiss-Harter model (2000), self-esteem is an antecedent of enjoyment, while enjoyment predicts physical activity.

In a recent article, the Weiss-Harter model was tested as a whole, allowing for the extrapolating of the multivariate associations among variables and their relevance in explaining physical activity in youth (Jekauc et al., 2019). In two samples of German participants aged 11–17 years, using cross-sectional data, structural equation modelling did not show a good model fit for the proposed model (in the smaller of the two samples, perceived competence was not included). This led the authors to compute an alternative model, where perceived social support also had a direct effect on enjoyment and physical activity (see Fig. 2 in the result section as a graphic representation). Because both parental and peer support are considered important sources for adolescents (Weiss, 2000), perceived social support was operationalised by a measure reflecting both sources (Reimers, Jekauc, Mess, Mewes, & Woll, 2012). This adapted model showed a better model fit in both samples than the original Weiss-Harter model (Jekauc et al., 2019). In particular, the adapted model showed strong direct effects of perceived social support on enjoyment and physical activity, whereas the direct effects of self-esteem on these variables were negligible. Thus, consistent with other research (Mendonça, Cheng, Mélo, & de Farias Júnior, 2014; Sallis, Prochaska, & Taylor, 2000), the authors concluded that perceived social support may play a more important role than initially assumed in the Weiss-Harter model (Jekauc et al., 2019).

## The current study

Given the decline in physical activity in adolescence, the Weiss-Harter model may provide a useful theoretical framework for understanding youth physical activity. While the Weiss-Harter model considers self-esteem as the most central construct mediating the effects of perceived competence and perceived social support (Weiss, 2000), two recent cross-sectional studies suggest that perceived social support may have direct effects on enjoyment and physical activity (Jekauc et al., 2019). Because the results of these studies were based on cross-sectional data, the purpose of the current study was to test the two alternative models in a prospective design. In particular, we tested and compared the extent to which the two models could predict (1) future physical activity and (2) a change in physical activity between the first and the second assessment.

## Methods

### Participants

The sample was derived from the Motorik-Modul-Study (MoMo; Woll et al., 2021), which is a submodule of the German Health Interview and Examination Survey for Children and Adolescents (KIGGS) conducted by the Robert Koch Institute. In both the MoMo- and the KIGGS-Study, participants were informed in detail about the study and gave written consent. Moreover, participants’ parents gave written consent for their children. The study was conducted according to the Declaration of Helsinki. Ethical approval was obtained by the Charité Universitätsmedizin Berlin ethics committee. For the KIGGS-Study, recruitment was conducted on the basis of a two-stage sampling approach (for a detailed description, see Kurth et al., 2008). In a first step, 167 communities were selected in Germany by proportionately considering the level of urbanization and geographic distribution (i.e., the communities were stratified according to the residence in former East Germany, West Germany, or the city of Berlin). In a second step, using an age-stratified procedure, children and adolescents aged 4–17 were randomly drawn from official registers. The MoMo-Study as a submodule of the KIGGs-Study aims to examine children’s and adolescents’ development of physical fitness and physical activity as well as their psychological, social, and environmental determinants (Woll et al., 2021). After the baseline assessment (T0: 2003–2006), three more assessment waves were conducted (T1: 2009–2012, T2: 2015–2017, and T3: 2018–2022). The present study investigated the associations between the assessments of T1 (2009–2012) and T2 (2015–2017). Baseline assessment (T0: 2003–2006) was not considered as data on the psychological constructs of interest was not collected for participants between the ages of 11 and 17 years. Also, we did not include data of T3 (2018–2022) as data collection partially took place before and partially during the Covid-19 pandemic. These different conditions of data collection could have potentially confounded our analysis. To assess the trajectory of physical activity, only those participants who took part in both assessment waves of interest (T1 and T2) and who were between 11 and 17 years old at T1 were considered. Subsequently, a total of 1107 participants (603 female) with a mean age of 13.98 (*SD* = 2.03) at T1 (wave 2009–2012) were considered for this study.

### Measures

For each individual, two measures of physical activity were considered. The first took place during the assessment wave T1 of 2009–2012 and the second during the assessment wave T2 of 2015–2017. For the four psychological constructs (i.e., perceived social support, perceived competence, self-esteem, and enjoyment), only the measures during the assessment wave T1 of 2009–2012 were considered.

#### Physical activity

The MoMo Physical Activity Questionnaire (Jekauc, Wagner, Kahlert, & Woll, 2013b) was used to measure physical activity. This questionnaire contains 28 items to measure physical activity in school, during leisure time, and in sports clubs. For those participants who were not at school anymore, physical activity at work was assessed. Previous results indicate a moderate test-retest reliability and a moderate correlation with accelerometer-recorded moderate-to-vigorous physical activity (MVPA; Jekauc et al., 2013b). In the present study, the outcome measure was minutes of MVPA per week.

#### Perceived social support (parental and peer)

Perceived social support was measured using two subscales of the German social support scale by Reimers et al. (2012). The first subscale includes 5 items (e.g., “Do your parents support you in your sport?”) measuring perceived support by parents and the second subscale includes 3 items (e.g., “How often do your friends ask you to play or to do sport together?”) measuring perceived support by peers on a 4-point Likert scale ranging from 1 = *never* to 4 = *always*. The questionnaire has been shown to have a moderate test-retest reliability, a good internal consistency and a moderate correlation with indices of physical activity (Reimers et al., 2012). In the present study, Cronbach’s alpha was 0.80 for perceived parental support and 0.78 for perceived peer support.

#### Perceived competence

Perceived competence was measured with the German version of the physical self-concept questionnaire (Stiller, Würth, & Alfermann, 2004), which is based on the hierarchical and multidimensional self-concept by Shavelson et al. (1976). Because the subscales of this questionnaire reflect perceptions of competence in different areas of physical activity (see examples below), this questionnaire was deemed suitable to measure the construct of perceived competence. Using a 4-point Likert scale from 1 = *does not apply* to 4 = *does apply*, this questionnaire includes 46 items divided into seven subscales. The subscale physical attractiveness (10 items) was not part of the data collection in the MoMo-Study and, therefore, this subscale was not included in the analysis. Thus, in the present study, the subscales strength (e.g., “I would be good in a test that measures strength.”), flexibility (e.g., “My body is flexible.”), endurance (e.g., “I’m good at endurance sports.”), speed (e.g., “I’m good in sports, in which you react and move quickly.”), coordination (e.g., “I’m good at coordinating my movements.”), and general sports competence (e.g., “Other people think I’m good in sports.”) were used. Each of the subscales consisted of 6 items. Previous results indicated that the subscales have a good internal consistency and that the questionnaire could distinguish between physically active and non-active children and adolescents (Stiller et al., 2004). Similar to the procedure of Vollmer, Lohmann, and Giess-Stüber (2021), we used the items of the six subscales to form item parcels as manifest indicators for the latent variable perceived competence. In the present study, Cronbach’s alpha was 0.90 for the subscale strength, 0.88 for the subscale flexibility, 0.89 for the subscale endurance, 0.83 for the subscale speed, 0.86 for the subscale coordination, and 0.90 for the subscale general sports competence.

#### Self-esteem

Four items were used for general self-esteem (e.g., “In general I’m happy with myself.”) and three items for physical self-esteem (e.g., “I like my body the way it is.”) answered on a 4-point Likert scale from 1 = *not true at all* to 4 = *exactly true*. These items have been previously used in the German Sprint-Study (Becker, 2006). The items for physical self-esteem were based on the physical self-worth subscale of the Physical Self-Perception Profile (Fox & Corbin, 1989) and the items for general self-esteem on the general self-esteem scale (Rosenberg, 1965), which is often used in conjunction with the Physical Self-Perception Profile. A previous study indicated good validity and reliability of the Physical Self-Perception Profile including the general self-esteem scale for children aged 9 years and older (Welk, Corbin, Dowell, & Harris, 1997). In the present study, Cronbach’s alpha was 0.86 for the subscale measuring general self-esteem and 0.81 for the subscale measuring physical self-esteem.

#### Enjoyment

Enjoyment was assessed with the short version of the Physical Activity Enjoyment Scale (PACES‑S; Chen et al., 2021). This recently published short form, which is based on items of the long version, made it possible to avoid the method effects for positively and negatively worded items associated with the long version of PACES (Jekauc, Voelkle, Wagner, Mewes, & Woll, 2013a). For this reason, the short form PACES‑S was used instead of the long version, which had been used in the data collection. The items (e.g., “I enjoy physical activity.”) were answered using a 5-point Likert scale ranging from 1 = *I disagree a lot* to 5 = *I agree a lot*. A previous study has shown a good test-retest-reliability and internal consistency as well as positive correlations with subjective and device-based measures of physical activity (Chen et al., 2021). In the present study, Cronbach’s alpha was 0.86.

#### Control variable

According to Weiss (2000), it is important to consider gender differences in how children and adolescence evaluate their competence in physical activity. For this reason, we controlled for gender in the following analyses (female = 1; male = 0).

### Statistical analysis

We used structural equation modelling in AMOS 26. Since the data had an interval scale level, we used full-information maximum likelihood estimation. This procedure provides less biased estimates with missing data than classical missing data procedures, such as list-/pairwise deletion or mean imputations (Jekauc, Völkle, Lämmle, & Woll, 2012). Little’s MCAR test indicated that the data were not missing completely at random (χ^2^ (4378) = 4755.23; *p* < 0.01). In the structural equation modelling, we tested and compared the extent to which the two models could predict (1) future physical activity at T2, and (2) a change in physical activity from T1 to T2. The psychological constructs were assessed at T1. For the first research question, MVPA at T2 was used as the dependent variable. Here, the original Weiss-Harter model is referred to below as “Model 1a” and the adapted model as “Model 1b”. For the second research question, we subtracted MVPA at T1 from MVPA at T2 for the dependent variable. Here, the original Weiss-Harter model is referred to below as “Model 2a” and the adapted model as “Model 2b”.

The overall fit of the models was assessed by χ^2^-statistic with a good model fit indicated by a non-significant *p*-value (Barrett, 2007). However, because this test is sensitive to large sample sizes (Hu & Bentler, 1999), the comparative fit index (CFI) as well as the root-mean-square error of approximation (RMSEA) were also considered. The CFI shows the relative improvement in fit by comparing the baseline model with the suggested model, with values between 0.90 and 0.95 indicating an acceptable fit and values above 0.95 indicating a good model fit (Hu & Bentler, 1999). The RMSEA shows the discrepancy between the suggested model with optimally selected parameters and the population covariance matrix. With regards to RMSEA, values between 0.05 and 0.08 indicate an acceptable model fit and values below 0.05 indicate a good model fit (Browne & Cudeck, 1992). The χ^2^-difference test was used to compare the two nested models for each research question, with a significant *p*-value indicating an improved model fit. Thus, Model 1a was compared to Model 1b for MVPA at the assessment t2 as the dependent variable and Model 2a was compared to Model 2b for the difference in MVPA between the two assessments (t2 − t1) as the dependent variable.

## Results

### Descriptive statistics

The descriptive statistics for the individual variables and the correlations between the individual variables are shown in Table 1. The correlations indicated positive associations between all psychological constructs of the first assessment as well as between the psychological constructs of the first assessment and MVPA of the second assessment. In addition, the results indicated that the difference of MVPA (t2 − t1) had a negative association with perceived peer support and endurance of the first assessment.

**Table 1:** Descriptive statistics of constructs and correlations between constructs

	Data available	Mean (SD)	Gender	Flexibility	Coordination	Strength	Speed	Endurance	Sports competence	Peer social support	Parents social support	Physical self-esteem	General self-esteem	Enjoy-ment	Weekly MVPA minutes t2	Weekly ∆MPVA minutes t2 − t1
Gender	1107 (100%)	–	–	–	–	–	–	–	–	–	–	–	–	–	–	–
Flexibility	1043 (94.2%)	3.10 (0.61)	−0.02	–	–	–	–	–	–	–	–	–	–	–	–	–
Coordination	1020 (92.1%)	3.10 (0.52)	−0.12**	0.67**	–	–	–	–	–	–	–	–	–	–	–	–
Strength	1023 (92.4%)	2.90 (0.65)	−0.29**	0.33**	0.42**	–	–	–	–	–	–	–	–	–	–	–
Speed	1044 (94.3%)	3.07 (0.59)	−0.21**	0.55**	0.67**	0.48**	–	–	–	–	–	–	–	–	–	–
Endurance	1043 (94.2%)	2.86 (0.71)	−0.31**	0.37**	0.53**	0.39**	0.53**	–	–	–	–	–	–	–	–	–
Sports Competence	1037 (93.7%)	3.09 (0.61)	−0.23**	0.55**	0.68**	0.56**	0.76**	0.61**	–	–	–	–	–	–	–	–
Peer social support	1091 (98.6%)	2.60 (0.58)	−0.21**	0.23**	0.28**	0.30**	0.29**	0.28**	0.39**	–	–	–	–	–	–	–
Parental social support	1065 (96.2%)	2.94 (0.57)	−0.11**	0.34**	0.42**	0.32**	0.35**	0.33**	0.46**	0.34**	–	–	–	–	–	–
Physical self-esteem	1054 (95.1%)	3.28 (0.57)	−0.17**	0.39**	0.44**	0.24**	0.41**	0.32**	0.40**	0.20**	0.31**	–	–	–	–	–
General self-esteem	1046 (94.5%)	3.15 (0.67)	−0.17**	0.37**	0.45**	0.33**	0.42**	0.35**	0.44**	0.24**	0.32**	0.72**	–	–	–	–
Enjoyment	1056 (95.4%)	4.03 (0.73)	−0.17**	0.38**	0.50**	0.37**	0.43**	0.42**	0.52**	0.38**	0.40**	0.33**	0.37**	–	–	–
Weekly MVPA minutes t2	1107 (100%)	233.63 (219.58)	−0.14**	0.19**	0.27**	0.25**	0.22*	0.27**	0.32**	0.19**	0.30**	0.16**	0.19**	0.27**	–	–
Weekly ∆MVPA minutes t2 − t1	1107 (100%)	−74.93 (233.73)	0.07*	−0.04	−0.04	−0.03	−0.07*	−0.03	−0.03	−0.08**	−0.01	0.02	−0.03	−0.04	0.61**	–

*SD* standard deviation, *MVPA* moderate-vigorous physical activity, *t1* first assessment, *t2* *−* *t1* difference between second and first assessment

* < 0.05; ** < 0.01

### MVPA at second assessment as dependent variable

**Fig. 1:**
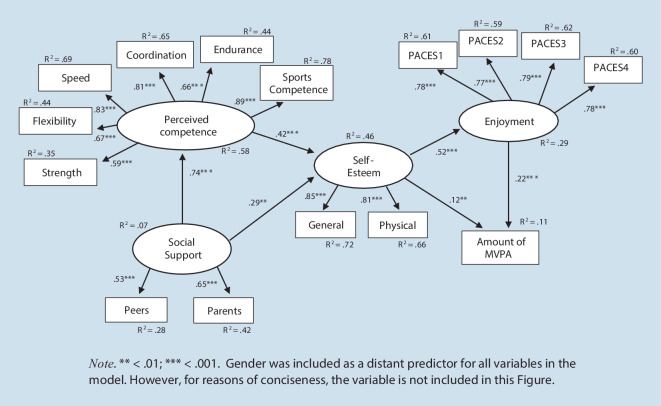
Original Weiss-Harter model: Model 1a

First, we assessed the two models Model 1a and Model 1b with the amount of MVPA of the second assessment as the dependent variable. All fit indices are shown in Table 2. For Model 1a, the *χ*^2^-statistic deviated significantly from zero. While the CFI indicated an acceptable model fit, the RMSEA indicated a poor model fit. As shown in Fig. 1, all associations between the constructs were significant. Self-esteem (β = 0.12, *SE* = 0.04, *p* < 0.001, 95% CI: 0.04, 0.20) and enjoyment (β = 0.22, *SE* = 0.04, *p* < 0.001, 95% CI: 0.14, 0.30) both had significant effects on the MVPA of the second assessment and together explained about 11% of its variance.

**Table 2:** Model fit indices

	χ^2^	*Df*	*p*	CFI	RMSEA	∆χ^2^	*∆df*	*p*
**MVPA t2**								
Model 1a	812.44	95	< 0.01	0.905	0.083	–	–	–
Model 1b	512.19	93	< 0.01	0.943	0.065	300.251	2	< 0.01
**∆MVPA t2** **−** **t1**								
Model 2a	755.29	95	< 0.01	0.910	0.079	–	–	–
Model 2b	513.25	93	< 0.01	0.943	0.064	242.04	2	< 0.01

*df* degrees of freedom, *p* probability value, *CFI* Comparative Fit Index, *RMSEA* Root Mean Square Error of Approximation, *∆χ*^*2*^ chi-square difference, *∆df* difference of degrees of freedom

**Fig. 2:**
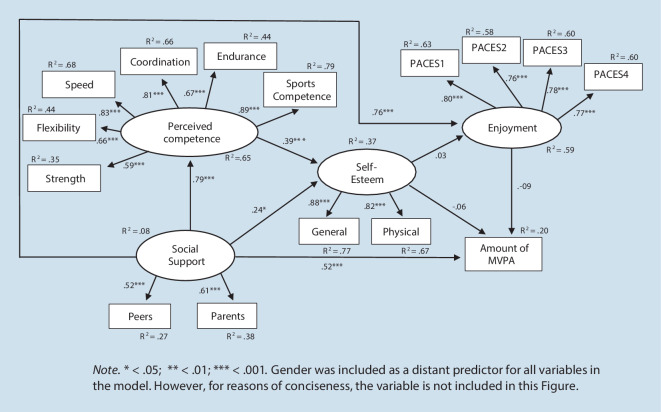
Adapted Weiss-Harter model: Model 1b

In Model 1b with two direct paths from perceived social support to enjoyment and MVPA, the model fit improved significantly. In Model 1b, the χ^2^-statistic also deviated significantly from zero, however, both CFI and RMSEA indicated an acceptable model fit. This finding supports the improved validity of the adapted model. The associations between the different constructs are shown in Fig. 2. In contrast to Model 1a, the direct effect of self-esteem on enjoyment as well as the direct effects of enjoyment and self-esteem on MVPA were no longer significant. However, perceived social support had a significant direct effect on both enjoyment (β = 0.76, *SE* = 0.08, *p* < 0.001, 95% CI: 0.59, 0.92) and MVPA (β = 0.52,* SE* = 0.10, *p* < 0.001, 95% CI: 0.33, 0.71). The amount of explained variance of MVPA at the second assessment was about 20%.

### Differences of MVPA from first to second assessment as dependent variable

**Fig. 3:**
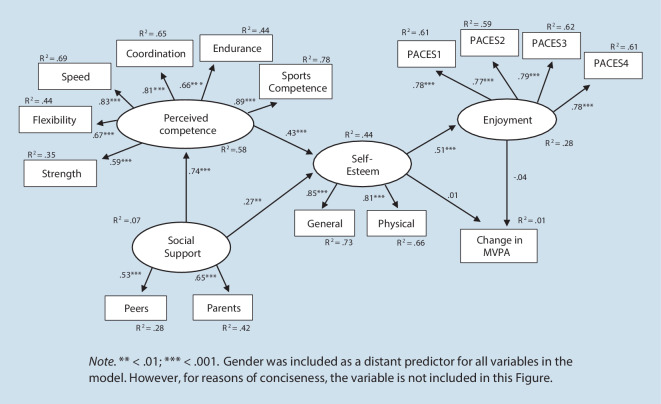
Original Weiss-Harter model: Model 2a

In a second step, we assessed Model 2a and Model 2b using the difference in MVPA between the first and the second assessment as the dependent variable. All fit indices are again shown in Table 2. For Model 2a, the χ^2^-statistic deviated significantly from zero. Both the CFI and RMSEA indicated an acceptable model fit. As shown in Fig. 3, with the exception of the direct effects of enjoyment and self-esteem on the difference in MVPA between the two assessments, all other associations were significant. The amount of explained variance of the difference of MVPA between the two assessments was 1%.

**Fig. 4:**
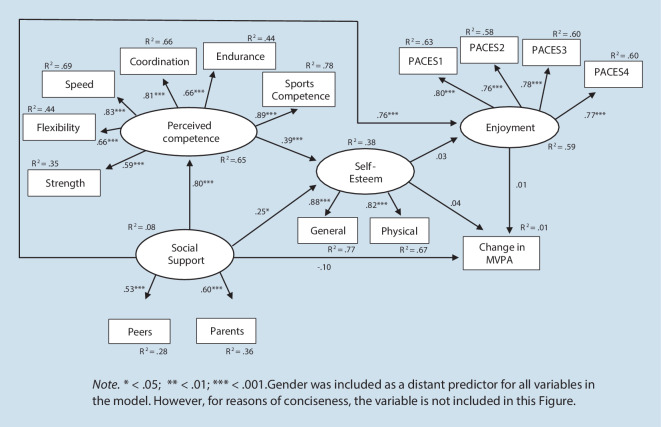
Adapted Weiss-Harter model: Model 2b

In Model 2b, with two direct paths from perceived social support to enjoyment and the difference in MVPA between the two assessments, the model fit improved significantly. For Model 2b, the *χ*2‑statistic deviated significantly from zero, however, both CFI and RMSEA indicated an acceptable model fit. The associations between the different constructs are shown in Fig. 4. Both self-esteem and enjoyment had no direct effects on the difference in MVPA between the two assessments. Moreover, there was no direct effect of self-esteem on enjoyment anymore. Perceived social support had a strong positive direct effect on enjoyment (β = 0.76, *SE* = 0.07, *p* < 0.001, 95% CI: 0.62, 0.90), however, a small non-significant negative direct effect (β = −0.10*, SE* = 0.09, *p* = 0.27, 95% CI: −0.27, 0.07) on the difference in MVPA between the two assessments. This negative direct effect indicated that a higher perceived social support at the first assessment was associated with a greater decline in MVPA from the first to the second assessment. The amount of explained variance of the difference of MVPA between the two assessments was 1%.

## Discussion

The Weiss-Harter model aims to contribute to a better understanding of physical activity in youth (Weiss, 2000). The model suggests that self-esteem mediates the effects of perceived competence and perceived social support on enjoyment and physical activity. However, two recent cross-sectional studies proposed an alternative model in which perceived social support has direct effects on enjoyment and physical activity (Jekauc et al., 2019). The purpose of the present study was to compare the two models in a prospective study design. The findings of the present study supported the adapted model.

The results of the present study showed for both indicators of MVPA, namely the level of MVPA of the second assessment and the change of MVPA from the first to the second assessment, a better model fit for the adapted model compared to the original Weiss-Harter model. Particularly, it was shown that the adapted model with direct effects of perceived social support on enjoyment and MVPA can explain about 20% of future MVPA. While this proportion of explained variance is about half the proportion of explained variance of the cross-sectional studies of Jekauc et al. (2019), it is twice as high as when the original Weiss-Harter model was applied in the present study. Thus, consistent with a previous cross-sectional study (Jekauc et al., 2019), the results suggest that perceived social support has a more important role than initially assumed by the Weiss-Harter model.

With regards to the role of self-esteem, in contrast to the cross-sectional study of Jekauc et al. (2019), in the original Weiss-Harter model with future MVPA as a dependent variable, self-esteem had a (albeit small) direct effect on MVPA (β = 0.12) in the present study. This finding is somewhat consistent with the theoretical assumption that considers self-esteem as an important determinant of behaviour (Shavelson et al., 1976; Weiss, 2000). However, given that self-esteem was measured only for the first assessment, it is important to consider the bi-directionality of the association between self-esteem and physical activity (e.g., Garn et al., 2019; Trautwein et al., 2008). Moreover, pointing to the importance of perceived social support, the effect of self-esteem on MVPA disappeared after adding the direct effects of perceived social support on enjoyment and MVPA.

The relevance of perceived social support for physical activity is consistent with previous research (for a systematic review on the association between physical activity and social support in adolescents see Mendonça et al., 2014). Regarding longitudinal effects, it was shown that instrumental support provided by family and peers was a significant predictor of children’s future MVPA (Siceloff, Wilson, & Van Horn, 2013). Focusing on girls across the ages of 9–15 years, in particular parental modelling (e.g., being active together with the child) and logistic support (e.g., enrolling child in activities) were identified as relevant factors for the maintenance of physical activity (Davison & Jago, 2009). Moreover, using a large sample with more than 850 children, it was shown that a higher perception of parental and peer support could mitigate the decline in physical activity typically found during adolescence (Dishman, Dowda, McIver, Saunders, & Pate, 2017).

In the present study, the results further showed that higher perceived social support for the first assessment was associated with a greater decline in MVPA. While this direct effect was small (*β* = −0.10) and the amount of total explained variance in this model was only 1%, this finding seems contra-intuitive at first. In this regard, it is important to keep in mind that various biological, psychological, and social changes potentially influence physical activity during adolescence. Thus, considering also the negative bivariate association between perceived peer support and the change of MVPA (t2 − t1), it may be that those individuals who had a high level of perceived social support at the time of the first assessment experienced a decrease in perceived social support, and this decrease negatively influenced their MVPA.

A review focusing on various correlates of physical activity suggested that male sex, self-efficacy, and previous physical activity were consistent correlates for all age groups (Bauman et al., 2012). However, reported health status and intention to exercise were consistent correlates of physical activity only for adults, while perceived social support from the family was a consistent correlate only for adolescents. The relevance of perceived social support for youth is in line with the finding of the present study and points to the importance of considering the different age groups when trying to better understand what leads a person to be physically active (Weiss, 2000). The fact that youth have typically less autonomy over their behaviour compared to adults underlines that it is particularly important for youth to have social settings that increase opportunities to be physically active provided for them (Rosenkranz et al., 2021).

Considering the practical implications of this study, the results indicate that interventions that intend to counteract the decline of MVPA during adolescence may in particular focus on perceived social support. In this regard, a meta-analysis showed that family-based interventions have a significant, albeit small, effect on MVPA (Brown et al., 2016). In this review, interventions employing goal-setting and reinforcement techniques were particularly highlighted as useful. This reasoning is consistent with a randomized control trial that incorporated constructs such as relatedness and goal-setting in their intervention to target the engagement of fathers in their daughters’ physical activity (Morgan et al., 2018). The results of this study indicate that this intervention could increase the daughters’ as well as the fathers’ physical activity, which was maintained at a follow-up after 9 months. The added benefit of involving both youth and parents in an intervention program is also supported by the study of Greening, Harrell, Low, and Fielder (2011). In this study, compared to the control group, in which students received the standard health curriculum in school, participants of the intervention group showed more physical activity and improvement in fitness tests after the intervention. Considering that perceived social support can also be increased from youth for youth, it is important to note that peer-delivered interventions have also been shown to increase MVPA, self-efficacy, and more autonomous forms of motivation (for a review see Ginis, Nigg, & Smith, 2013). Such peer-delivered interventions may be especially an alternative when parents have limited capacities (e.g., due to work constraints), pointing to the relevance of tailoring the content of such an intervention to the needs of the involved persons.

## Limitations

There are several limitations that should be considered when interpreting the results of the present study. First, although the prospective study design allows extrapolation of the temporal effects of the psychological constructs on future MVPA, with the inclusion of only two measurement occasions, only linear trends can be assumed. Because this does not necessarily represent the true development pattern, it is important to include more than two measurement points in order to be able to make more valid statements on longitudinal trajectories (Singer & Willett, 2003). Moreover, the psychological constructs were only based on one assessment and it is reasonable to assume that there have been profound changes in these constructs up to the time of the second assessment. In addition, there might also have been other psychological constructs (e.g., habit; Feil, Allion, Weyland, & Jekauc, 2021) or environmental aspects (e.g., population density; Nigg et al., 2021) influencing MVPA that we did not control for. Moreover, although the use of questionnaires to assess MVPA appears to be beneficial for studies with large sample sizes (Nigg et al., 2020), the use of device-based methods could have reduced potential biases of such methods. Furthermore, it is important to mention that the construct of perceived competence was measured by a questionnaire, which intends to measure physical self-concept (Stiller et al., 2004), and thus might not have been specific enough for perceived competence. In this regard, in the data collection of the MoMo study, the physical attractiveness subscale of the Physical Self-Concept Questionnaire (Stiller et al., 2004) was not included and therefore could not be taken into account in the assessment of the psychological construct of perceived competence. Considering that the Weiss-Harter model explicitly defines perceived competence as “children assessing how adequate they are in the areas of sport, physical attractiveness and physical fitness” (Weiss, 2000; p. 2), it is important to include this subscale in future studies. It is also notable that missing data were not completely at random, which could have affected the results. Finally, the large representative sample size of the MoMo-Study allows generalizing the findings across Germany. However, as in the previous cross-sectional study (Jekauc et al., 2019), the results are limited to the German population, pointing to the need to test the models in other countries.

## Conclusion

The present study supports the adapted version of the Weiss-Harter model. In particular, consistent with cross-sectional findings (Jekauc et al., 2019), the results of this study indicate that perceived social support has direct effects on enjoyment and future physical activity. Given the decline in physical activity often seen in adolescence, we encourage, based on the findings, that future research focuses particularly on how to increase the perceived social support of children and adolescents.

## Declarations

All procedures performed in studies involving human participants or on human tissue were in accordance with the ethical standards of the institutional and/or national research committee and with the 1975 Helsinki declaration and its later amendments or comparable ethical standards. Informed consent was obtained from all individual participants or their legal guardians.

## References

[CR1] Adachi, P. J., & Willoughby, T. (2014). It’s not how much you play, but how much you enjoy the game: the longitudinal associations between adolescents’ self-esteem and the frequency versus enjoyment of involvement in sports. *Journal of Youth and Adolescence*, *43*, 137–145. 10.1007/s10964-013-9988-3. 23933963 10.1007/s10964-013-9988-3

[CR2] Ajzen, I. (1991). The theory of planned behavior. *Organizational Behavior and Human Decision Processes*, *50*, 179–211. 10.1016/0749-5978(91)90020-T.

[CR3] Babic, M. J., Morgan, P. J., Plotnikoff, R. C., Lonsdale, C., White, R. L., & Lubans, D. R. (2014). Physical activity and physical self-concept in youth: Systematic review and meta-analysis. *Sports Medicine*, *44*, 1589–1601. 10.1007/s40279-014-0229-z. 25053012 10.1007/s40279-014-0229-z

[CR5] Barrett, P. (2007). Structural equation modelling: Adjudging model fit. *Personality and Individual Differences*, *42*, 815–824. 10.1016/j.paid.2006.09.018.

[CR4] Bauman, A. E., Reis, R. S., Sallis, J. F., Wells, J. C., Loos, R. J., Martin, B. W., & Lancet Physical Activity Series Working Group (2012). Correlates of physical activity: why are some people physically active and others not? *Lancet*, *380*, 258–271. 10.1016/S0140-6736(12)60735-1. 22818938 10.1016/S0140-6736(12)60735-1

[CR6] Becker, J. (2006). *DSB-Sprint-Studie: Eine Untersuchung zur Situation des Schulsports in Deutschland [DSB-Sprint-Study: An investigation on the situation of physical education in Germany]*. Meyer & Meyer.

[CR7] Brand, R., & Ekkekakis, P. (2018). Affective–reflective theory of physical inactivity and exercise. *German Journal of Exercise and Sport Research*, *48*, 48–58. 10.1007/s12662-017-0477-9.

[CR8] Brown, H. E., Atkin, A. J., Panter, J., Wong, G., Chinapaw, M. J. M., & van Sluijs, E. M. F. (2016). Family-based interventions to increase physical activity in children: a systematic review, meta-analysis and realist synthesis. *Obesity Reviews*, *17*, 345–360. 10.1111/obr.12362. 26756281 10.1111/obr.12362 PMC4819691

[CR9] Browne, M. W., & Cudeck, R. (1992). Alternative ways of assessing model fit. *Sociological Methods & Research*, *21*, 230–258. 10.1177/0049124192021002005.

[CR10] Chen, C., Weyland, S., Fritsch, J., Woll, A., Niessner, C., Burchartz, A., Schmidt, S. C. E., & Jekauc, D. (2021). A short version of the physical activity enjoyment scale: Development and psychometric properties. *International Journal of Environmental Research and Public Health*, *18*, 11035. https://www.mdpi.com/1660-4601/18/21/11035. 34769552 10.3390/ijerph182111035 PMC8582913

[CR11] Davison, K. K., & Jago, R. (2009). Change in parent and peer support across ages 9 to 15 yr and adolescent girls’ physical activity. *Medicine and Science in Sports and Exercise*, *41*, 1816–1825. 10.1249/MSS.0b013e3181a278e2. 19657287 10.1249/MSS.0b013e3181a278e2 PMC5489408

[CR12] deJonge, M., Mackowiak, R., Pila, E., Crocker, P. R., & Sabiston, C. M. (2019). The relationship between sport commitment and physical self-concept: Evidence for the self-enhancement hypothesis among adolescent females. *Journal of Sports Sciences*, *37*, 2459–2466. 10.1080/02640414.2019.1641381. 31288678 10.1080/02640414.2019.1641381

[CR13] Dishman, R. K., Dowda, M., McIver, K. L., Saunders, R. P., & Pate, R. R. (2017). Naturally-occurring changes in social-cognitive factors modify change in physical activity during early adolescence. *PLoS ONE*, *12*, e172040. 10.1371/journal.pone.0172040. 28187192 10.1371/journal.pone.0172040 PMC5302819

[CR14] Ebbeck, V., & Weiss, M. R. (1998). Determinants of children’s self-esteem: an examination of perceived competence and affect in sport. *Pediatric Exercise Science*, *10*, 285–298. 10.1123/pes.10.3.285.

[CR15] Ekblom-Bak, E., Ekblom, Ö., Andersson, G., Wallin, P., & Ekblom, B. (2018). Physical education and leisure-time physical activity in youth are both important for adulthood activity, physical performance, and health. *Journal of Physical Activity and Health*, *15*, 661–670. 10.1123/jpah.2017-0083. 29706117 10.1123/jpah.2017-0083

[CR16] Feil, K., Allion, S., Weyland, S., & Jekauc, D. (2021). A systematic review examining the relationship between habit and physical activity behavior in longitudinal studies. *Frontiers in Psycholgy*, *12*, 626750. 10.3389/fpsyg.2021.626750. 10.3389/fpsyg.2021.626750 PMC7969808 33746848

[CR17] Finne, E., Bucksch, J., Lampert, T., & Kolip, P. (2011). Age, puberty, body dissatisfaction, and physical activity decline in adolescents. Results of the German Health Interview and Examination Survey (KiGGS). *International Journal of Behavioral Nutrition and Physical Activity*, *8*, 119. 10.1186/1479-5868-8-119. 22032266 10.1186/1479-5868-8-119 PMC3231807

[CR18] Fitzgerald, A., Fitzgerald, N., & Aherne, C. (2012). Do peers matter? A review of peer and/or friends’ influence on physical activity among American adolescents. *Journal of Adolescence*, *35*, 941–958. 10.1016/j.adolescence.2012.01.002. 22285398 10.1016/j.adolescence.2012.01.002

[CR19] Fox, K. R., & Corbin, C. B. (1989). The physical self-perception profile: development and preliminary validation. *Journal of Sport & Exercise Psychology*, *11*, 408–430.

[CR20] Fox, K. R., & Wilson, P. M. (2008). Self-perceptual systems and physical activity. In T. S. Horn (Ed.), *Advances in sport psychology* (pp. 49–62). Human Kinetics.

[CR21] Garn, A. C., Moore, E. W., Centeio, E. E., Kulik, N., Somers, C., & McCaughtry, N. (2019). Reciprocal effects model of children’s physical activity, physical self-concept, and enjoyment. *Psychology of Sport and Exercise*, *45*, 101568. 10.1016/j.psychsport.2019.101568. 10.1123/jsep.2015-0255 27385738

[CR22] Garriguet, D., Colley, R., & Bushnik, T. (2017). Parent-child association in physical activity and sedentary behaviour. *Health Reports*, *28*, 3–11. 28636068

[CR24] Ginis, K. A., Nigg, C. R., & Smith, A. L. (2013). Peer-delivered physical activity interventions: an overlooked opportunity for physical activity promotion. *Translational Behavioral Medicine*, *3*, 434–443. 10.1007/s13142-013-0215-2. 24294332 10.1007/s13142-013-0215-2 PMC3830020

[CR23] Greening, L., Harrell, K. T., Low, A. K., & Fielder, C. E. (2011). Efficacy of a school-based childhood obesity intervention program in a rural southern community: TEAM Mississippi Project. *Obesity*, *19*, 1213–1219. 10.1038/oby.2010.329. 21233806 10.1038/oby.2010.329

[CR25] Haas, P., Yang, C.-H., & Dunton, G. F. (2021). Associations between physical activity enjoyment and age-related decline in physical activity in children—results from a longitudinal within-person study. *Journal of Sport & Exercise Psychology*, *43*, 205–214. 10.1123/jsep.2020-0156. 33811189 10.1123/jsep.2020-0156

[CR26] Hagger, M. S., Chatzisarantis, N. L. D., & Biddle, S. J. H. (2002). A meta-analytic review of the theories of reasoned action and planned behavior in physical activity: predictive validity and the contribution of additional variables. *Journal of Sport & Exercise Psychology*, *24*, 3–32. 10.1123/jsep.24.1.3.

[CR27] Harter, S. (1978). Effectance motivation reconsidered: toward a developmental model. *Human Development*, *21*, 34–64. 10.1159/000271574.

[CR28] Harter, S. (1987). The determinants and mediational role of global self-worth in children. In N. Eisenberg (Ed.), *Contemporary Topics in Developmental Psychology* (pp. 219–242). John Wiley.

[CR29] Hu, L., & Bentler, P. M. (1999). Cutoff criteria for fit indexes in covariance structure analysis: conventional criteria versus new alternatives. *Structural Equation Modeling: A Multidisciplinary Journal*, *6*, 1–55. 10.1080/10705519909540118.

[CR31] Jekauc, D., Völkle, M., Lämmle, L., & Woll, A. (2012). Fehlende Werte in sportwissenschaftlichen Untersuchungen. *Sportwissenschaft*, *42*, 126–136. 10.1007/s12662-012-0249-5.

[CR32] Jekauc, D., Voelkle, M., Wagner, M. O., Mewes, N., & Woll, A. (2013a). Reliability, validity, and measurement invariance of the German version of the physical activity enjoyment scale. *Journal of Pediatric Psychology*, *38*, 104–115. 10.1093/jpepsy/jss088. 22946084 10.1093/jpepsy/jss088

[CR33] Jekauc, D., Wagner, M. O., Kahlert, D., & Woll, A. (2013b). Reliabilität und Validität des MoMo-Aktivitätsfragebogens für Jugendliche (MoMo-AFB). *Diagnostica*, *59*, 100–111. 10.1026/0012-1924/a000083.

[CR30] Jekauc, D., Mnich, C., Niessner, C., Wunsch, K., Nigg, C. R., Krell-Roesch, J., & Woll, A. (2019). Testing the Weiss-Harter-model: physical activity, self-esteem, enjoyment, and social support in children and adolescents. *Frontiers in Psychology*. 10.3389/fpsyg.2019.02568. 31803111 10.3389/fpsyg.2019.02568 PMC6872523

[CR34] Kandola, A., Lewis, G., Osborn, D. P. J., Stubbs, B., & Hayes, J. F. (2020). Depressive symptoms and objectively measured physical activity and sedentary behaviour throughout adolescence: a prospective cohort study. *The Lancet Psychiatry*, *7*, 262–271. 10.1016/S2215-0366(20)30034-1. 32059797 10.1016/S2215-0366(20)30034-1 PMC7033559

[CR35] Kurth, B. M., Kamtsiuris, P., Hölling, H., Schlaud, M., Dölle, R., Ellert, U., Kahl, H., Knopf, H., Lange, M., Mensink, G. B., Neuhauser, H., Rosario, A. S., Scheidt-Nave, C., Schenk, L., Schlack, R., Stolzenberg, H., Thamm, M., Thierfelder, W., & Wolf, U. (2008). The challenge of comprehensively mapping children’s health in a nation-wide health survey: design of the German KiGGS-Study. *BMC Public Health*, *8*, 196. 10.1186/1471-2458-8-196. 18533019 10.1186/1471-2458-8-196 PMC2442072

[CR36] Luna, B., Padmanabhan, A., & O’Hearn, K. (2010). What has fMRI told us about the development of cognitive control through adolescence? *Brain and Cognition*, *72*, 101–113. 10.1016/j.bandc.2009.08.005. 19765880 10.1016/j.bandc.2009.08.005 PMC2815087

[CR37] Mantovani, A. M., de Lima, M. C. S., Gobbo, L. A., Ronque, E. R. V., Romanzini, M., Turi-Lynch, B. C., Codogno, J. S., & Fernandes, R. A. (2018). Adults engaged in sports in early life have higher bone mass than their inactive peers. *Journal of Physical Activity and Health*, *15*, 516–522. 10.1123/jpah.2017-0366. 29569996 10.1123/jpah.2017-0366

[CR39] Marsh, H. W., & Redmayne, R. S. (1994). A multidimensional physical self-concept and its relations to multiple components of physical fitness. *Journal of Sport & Exercise Psychology*, *16*, 43–55.

[CR38] Marsh, H. W., Papaioannou, A., & Theodorakis, Y. (2006). Causal ordering of physical self-concept and exercise behavior: reciprocal effects model and the influence of physical education teachers. *Health Psychology*, *25*, 316–328. 10.1037/0278-6133.25.3.316. 16719603 10.1037/0278-6133.25.3.316

[CR40] Mendonça, G., Cheng, L. A., Mélo, E. N., & de Farias Júnior, J. C. (2014). Physical activity and social support in adolescents: a systematic review. *Health Education Research*, *29*, 822–839. 10.1093/her/cyu017. 24812148 10.1093/her/cyu017

[CR41] Morgan, P. J., Young, M. D., Barnes, A. T., Eather, N., Pollock, E. R., & Lubans, D. R. (2018). Engaging fathers to increase physical activity in girls: the “Dads And Daughters Exercising and Empowered” (DADEE) randomized controlled trial. *Annals of Behavioral Medicine*, *53*, 39–52. 10.1093/abm/kay015. 10.1093/abm/kay015 29648571

[CR42] Nasuti, G., & Rhodes, R. E. (2013). Affective judgment and physical activity in youth: review and meta-analyses. *Annals of Behavioral Medicine*, *45*, 357–376. 10.1007/s12160-012-9462-6. 23297073 10.1007/s12160-012-9462-6

[CR43] Nigg, C., Oriwol, D., Wunsch, K., Burchartz, A., Kolb, S., Worth, A., Woll, A., & Niessner, C. (2021). Population density predicts youth’s physical activity changes during Covid-19—results from the MoMo study. *Health & Place*, *70*, 102619. 10.1016/j.healthplace.2021.102619. 34233210 10.1016/j.healthplace.2021.102619 PMC9190022

[CR44] Nigg, C. R., Fuchs, R., Gerber, M., Jekauc, D., Koch, T., Krell-Roesch, J., Lippke, S., Mnich, C., Novak, B., Ju, Q., Sattler, M. C., Schmidt, S. C. E., van Poppel, M., Reimers, A. K., Wagner, P., Woods, C., & Woll, A. (2020). Assessing physical activity through questionnaires—A consensus of best practices and future directions. *Psychology of Sport and Exercise*, *50*, 101715. 10.1016/j.psychsport.2020.101715.

[CR45] Reimers, A. K., Jekauc, D., Mess, F., Mewes, N., & Woll, A. (2012). Validity and reliability of a self-report instrument to assess social support and physical environmental correlates of physical activity in adolescents. *BMC Public Health*, *12*, 705. 10.1186/1471-2458-12-705. 22928865 10.1186/1471-2458-12-705 PMC3489617

[CR46] Rhodes, R. E., & Kates, A. (2015). Can the affective response to exercise predict future motives and physical activity behavior? A systematic review of published evidence. *Annals of Behavioral Medicine*, *49*, 715–731. 10.1007/s12160-015-9704-5. 25921307 10.1007/s12160-015-9704-5

[CR47] Rosenberg, M. (1965). *Society and the adolescent self-image*. Princeton University Press.

[CR48] Rosenkranz, R. R., Ridley, K., Guagliano, J. M., & Rosenkranz, S. K. (2021). Physical activity capability, opportunity, motivation and behavior in youth settings: Theoretical framework to guide physical activity leader interventions. *International Review of Sport and Exercise Psychology*. 10.1080/1750984X.2021.1904434.

[CR49] Sallis, J. F., Prochaska, J. J., & Taylor, W. C. (2000). A review of correlates of physical activity of children and adolescents. *Medicine and Science in Sports and Exercise*, *32*, 963–975. 10.1097/00005768-200005000-00014. 10795788 10.1097/00005768-200005000-00014

[CR50] Shavelson, R. J., Hubner, J. J., & Stanton, G. C. (1976). Self-concept: Validation of construct interpretations. *Review of Educational Research*, *46*, 407–411.

[CR51] Siceloff, E. R., Wilson, D. K., & Van Horn, L. (2013). A longitudinal study of the effects of instrumental and emotional social support on physical activity in underserved adolescents in the ACT trial. *Annals of Behavioral Medicine*, *48*, 71–79. 10.1007/s12160-013-9571-x. 10.1007/s12160-013-9571-x PMC4137464 24327135

[CR52] Silva, P., Lott, R., Mota, J., & Welk, G. (2014). Direct and indirect effects of social support on youth physical activity behavior. *Pediatric Exercise Science*, *26*, 86–94. 10.1123/pes.2012-0207. 24018255 10.1123/pes.2012-0207

[CR53] Singer, J. D., & Willett, J. B. (2003). *Applied longitudinal data analysis: Modeling change and event occurrence*. Oxford University Press.

[CR54] Sonstroem, R. J., & Morgan, W. P. (1989). Exercise and self-esteem: rationale and model. *Medicine and Science in Sports and Exercise*, *21*, 329–337. 10.1249/00005768-198906000-00018. 2659918

[CR55] Stiller, J., Würth, S., & Alfermann, D. (2004). Die Messung des physischen Selbstkonzepts (PSK) [The Measurement of Physical Self-Concept (PSK)—The Development of the PSK-Scales for Children, Adolescents, and Young Adults]. *Zeitschrift für differentielle und diagnostische Psychologie*, *25*, 239–257. 10.1024/0170-1789.25.4.239.

[CR56] Strobach, T., Englert, C., Jekauc, D., & Pfeffer, I. (2020). Predicting adoption and maintenance of physical activity in the context of dual-process theories. *Performance Enhancement & Health*, *8*, 100162. 10.1016/j.peh.2020.100162.

[CR57] Tate, E. B., Shah, A., Jones, M., Pentz, M. A., Liao, Y., & Dunton, G. (2015). Toward a better understanding of the link between parent and child physical activity levels: the moderating role of parental encouragement. *Journal of Physical Activity and Health*, *12*, 1238–1244. 10.1123/jpah.2014-0126. 25494399 10.1123/jpah.2014-0126 PMC5504529

[CR58] Telama, R., Yang, X., Leskinen, E., Kankaanpää, A., Hirvensalo, M., Tammelin, T., Viikari, J. S., & Raitakari, O. T. (2014). Tracking of physical activity from early childhood through youth into adulthood. *Medicine and Science in Sports and Exercise*, *46*, 955–962. 10.1249/mss.0000000000000181. 24121247 10.1249/MSS.0000000000000181

[CR59] Trautwein, U., Gerlach, E., & Ludtke, O. (2008). Athletic classmates, physical self-concept, and free-time physical activity: a longitudinal study of frame of reference effects. *Journal of Educational Psychology*, *100*, 988–1001. 10.1037/0022-0663.100.4.988.

[CR60] Vollmer, J., Lohmann, J., & Giess-Stüber, P. (2021). Socioeconomic status and global physical self-concept of adolescents: a multilevel structural equation modeling approach. *German Journal of Exercise and Sport Research*, *51*, 160–169. 10.1007/s12662-020-00701-7.

[CR61] Wagnsson, S., Lindwall, M., & Gustafsson, H. (2014). Participation in organized sport and self-esteem across adolescence: the mediating role of perceived sport competence. *Journal of Sport and Exercise Psychology*, *36*, 584–594. 10.1123/jsep.2013-0137. 25602141 10.1123/jsep.2013-0137

[CR62] Weiss, M. R. (2000). Motivating kids in physical activity. *President’s Council on Physical Fitness and Sports Research Digest*, *3*, 1–8. PMC3022443 21253445

[CR63] Welk, G. J., Corbin, C. B., Dowell, M. N., & Harris, H. (1997). The validity and reliability of two different versions of the children and youth Physical Self-Perception Profile. *Measurement in Physical Education and Exercise Science*, *1*, 163–177. 10.1207/s15327841mpee0103_2.

[CR64] Woll, A., Klos, L., Burchartz, A., Hanssen-Doose, A., Niessner, C., Oriwol, D., Schmidt, S. C. E., Bös, K., & Worth, A. (2021). Cohort profile update: the Motorik-Modul (MoMo) longitudinal study-physical fitness and physical activity as determinants of health development in German children and adolescents. *International Journal of Epidemiology*, *50*, 393–394. 10.1093/ije/dyaa281. 33709121 10.1093/ije/dyaa281

